# The endothelial lipase protein is promising urinary biomarker for diagnosis of gastric cancer

**DOI:** 10.1186/1746-1596-8-45

**Published:** 2013-03-19

**Authors:** Xueyan Dong, Guoqing Wang, Guoqing Zhang, Zhaohui Ni, Jian Suo, Juan Cui, Ai Cui, Qing Yang, Ying Xu, Fan Li

**Affiliations:** 1 Department of Pathogeny Biology, Norman Bethune Medical College of Jilin University, 126 Xinmin street, Changchun, Jilin, 130021, China; 2 Cancer Hospital Affiliated with Xinjiang Medical University, Urumqi, Xinjiang, 830000, China; 3 The First Hospital of Jilin University Norman Bethune Medical College, Changchun, Jilin, 130021, China; 4 Computational Systems Biology Laboratory, Department of Biochemistry and Molecular Biology, and Institute of Bioinformatics, University of Georgia, Athens, GA, 30602-7229, USA; 5 College of Computer Science and Technology, Jilin University, Changchun, Jilin, 130012, China

**Keywords:** Endothelial lipase, Biomarker, Gastric cancer, Diagnosis

## Abstract

**Background:**

Gastric cancer is one of the most common malignant tumors in the world. Finding effective diagnostic biomarkers in urine or serum would represent the most ideal solution to detecting gastric cancer during annual physical examination. This study was to evaluate the potential of endothelial lipase (EL) as a urinary biomarker for diagnosis of gastric cancer.

**Methods:**

The expression levels of EL was measured using Western blotting and immunohistochemical staining experiments on (tissue, serum, and urine) samples of gastric cancer patients *versus* healthy people. We also checked the EL levels in the urine samples of other cancer types (lung, colon and rectum cancers) and benign lesions (gastritis and gastric leiomyoma) to check if EL was specific to gastric cancer.

**Result:**

We observed a clear separation between the EL expression levels in the urine samples of 90 gastric cancer patients and of 57 healthy volunteers. It was approximately 9.9 fold average decrease of the EL expression levels in the urine samples of gastric cancer compared to the healthy controls (*P* <0.0001), achieving a 0.967 AUC value for the ROC (receiver operating characteristic) curve, demonstrating it’s highly accurate as a diagnostic marker for gastric cancer. Interestingly, the expression levels of EL in tissue and serum samples were not nearly as discriminative as in urine samples (*P* = 0.90 and *P* = 0.79). In immunohistochemical experiments, positive expression of the EL protein was found in 67% (8/12) of gastric adjacent noncancerous and in 58% (7/12) of gastric cancer samples. There was no significant statistical in the expression levels of this protein between the gastric cancer and the matching noncancerous tissues (*P* =0.67).

**Conclusions:**

The urinary EL as a highly accurate gastric cancer biomarker that is potentially applicable to the general screening with high sensitivity and specificity.

**Virtual Slides:**

The virtual slide(s) for this article can be found here: http://www.diagnosticpathology.diagnomx.eu/vs/4527331618757552

## Background

Gastric cancer is one of the most common malignant tumors in the world, representing the third leading cause for cancer-related death in men and the fifth leading cause in women [1]. Approximately two-thirds of gastric cancer cases occur in less developed countries, which are almost three times higher per capita than in the developed countries, such as European countries and North America [2]. Gastric cancer has high mortality rates since it has no obvious clinical symptoms in the early stage. Studies suggest that if the tumor was detected and resected at the early stage, the average 5-year survival rate is relatively high [3,4]. Therefore, early diagnosis and treatment represent a key to improving the prognosis of gastric cancer patients.

Although great amount of effort has been put into the technology development to facilitate diagnosis using gastroscopy and immunohistochemical analysis, the invasive nature of these procedures makes it impractical for large-scale screening for gastric cancer. Effective diagnostic biomarkers in urine or serum would represent the most ideal solution to the problem, which allows testing for gastric cancer through blood or urine tests during annual physical examinations. Several diagnostic serum markers have been proposed for gastric cancer, such as MG7-Ag [5], carcinoembryonic antigen (CEA), MUC1 and MUC5AC [6]. The state of the art is that virtually all of them suffer from rather low sensitivities and specificities in cancer diagnosis, and hence have not been widely used clinically [7]. Compared with tissue and serum, urine collection is relatively easier and less invasive. It may be more suitable for large scale screening for cancer detection [8]. Using comparative proteomic analyses, a number of potential urinary biomarkers have been proposed and well tested for other types of cancers, such as cystatin B and clusterin for bladder cancer [9,10], carbonic anhydrase IX and cathepsin D for renal cancer [11,12], and ADAM12 for breast cancer [13]. For gastric cancer, several biomarkers have been reported, such as pepsinogen I, prostaglandin E2, and soluble c-erbB-2 [14-16]. The actual diagnostic ability for gastric cancer is yet to be thoroughly assessed, as they have not been widely used in clinical diagnostic. Therefore, there is clearly an urgent need for identification and validation of novel and reliable diagnostic markers for screening gastric cancer.

We have previously carried out a system biology study to identify potential candidate biomarkers for diagnosis of gastric cancer. In that study, we analyzed the gene-expression data of 80 pairs of gastric cancer tissues and adjacent non-cancerous tissues collected from gastric cancer patients using microarray chips, and identified hundreds of differentially expressed genes in cancer *versus* control tissues [17]. We then trained a support vector machine (SVM) based classifier to predict which of these differentially expressed genes may have their proteins excreted into urine. Among the predicted excretory protein, we found EL shows high discriminating power in terms of its expression in urine samples of gastric cancer patients *versus* healthy people [18].

Here we extend our previous study aiming to (a) further confirm that EL has high discriminative power between urine samples of gastric cancer patients and healthy people over a larger set of samples, and (b) demonstrate that EL is highly specific to gastric cancer by comparing its abundances in urine samples of gastric cancer with other cancer types and benign lesions.

## Methods

### Sample collection

All samples were collected at three hospitals of Jilin University Norman Bethune Medical College, Changchun, China and at the Cancer Hospital affiliated with Xinjiang Medical University, Urumqi, China. The gastric cancer tissues and the matching non-cancerous tissues were surgically resected from gastric cancer patients. The serum and morning random-catch urine samples were obtained from the cancer patients before surgery. These specimen samples were collected, during the period from March 2011 to September 2012. A total of 90 gastric cancer cases (67males and 23 females; age range: 31–85 years) and 57 healthy volunteers (30 males and 27 females; age range: 29–76 years) were studied. The gastric cancer cases were diagnosed by histological analyses as intestinal and diffuse type according to Lauren classification. In addition, urine samples were obtained from 9 lung cancer patients, 10 from colon cancer patients, 10 rectum cancer patients, 2 gastritis patients, and 2 gastric leiomyoma patients, which are used to check if EL is specific to gastric cancer. The following criteria were used in the sample collection: (i) cancer patients should not have started any treatment on their cancer; and (ii) volunteers in the healthy control group should not have any serious systemic disease in the past. A written informed consent form was signed by each participant after they were informed about the purpose of the study, which was approved by the Research Ethics Committees at Jilin University College of Medicine and Xinjiang Medical University, respectively. Table 1 summarizes the information of the healthy volunteers and patients involved in this study.

**Table 1:** The information of healthy volunteers and patients involved in this study

**Characteristics**	**Tissue**	**Serum**	**Urine**
**Gastric cancer**	12	12	90
Mean age (range)	60(42–76)	63(84–39)	60(31–85)
Gender			
Male	6	7	67
Female	6	5	23
Tumor location			
Cardia	3	2	39
Body	5	6	26
Antrum	3	4	16
Diffuse	1	0	9
Operation			
Total Gastrectomy	4	5	29
Subtotal Gastrectomy	8	7	61
Lauren classification			
Intestinal	5	4	39
Diffuse	7	8	51
Histology type			
High or moderate	5	5	43
Poor or undifferentiated	7	7	47
Tumor stages			
Ι and ΙΙ	2	4	31
ΙΙΙ and ΙV	10	8	59
Depth of invasion			
T1 and T2	5	9	30
T3 and T4	7	3	60
Node status			
N0	3	3	29
N1 and N2	9	9	61
Metastasis status			
M0	5	10	71
M1	7	2	19
**Lung cancer**	-	-	9
Mean age (range)			46(42–57)
**Colon cancer**	-	-	10
Mean age (range)			53(46–65)
**Rectum cancer**	-	-	9
Mean age (range)			62(47–78)
**Healthy**	12	12	57
Mean age (range)	60(42–76)	54(41–72)	47 (29–76)
**Gastritis/Chronic inflammation**	-	-	2
Mean age (range)			56 (43–69)
**Gastritic leiomyoma**			2
Mean age (range)	-	-	56 (51–62)

### Western blotting analysis

The tissues were grinded to powder in liquid nitrogen and then lysed in protein extraction buffer [0.5 mol/L Tris · Cl (pH 7.4), 150 mmol/L NaCl, 0.1 mmol/L ethylene diamine tetra aceticacid (EDTA) (pH 7.0), 1 mmol/L phenylmethylsulfonyl fluoride (PMSF), 2.5 mg/mL aprotinin, 1 mmol/L dithiothreitol (DTT), 1% Triton X-100, 1% sodium deoxycholate (SDS)]. All reagents were purchased from Beyotime (Beyotime, Shanghai, China). Serum and urine samples were stored in the presence of protease inhibitor (Roche, Basel, Switzerland) sterile containers and centrifuged (1,000 × g for 10 minutes at 4°C) to remove cellular components. The supernatants were collected and stored at −80°C (the longest storage for 6 months). 2 ml urine samples were dialyzed against distilled water through a filtration membrane (Dinguo, Beijing, China) of 8 kDa cutoff at 4°C and then lyophilized at −20°C. Freeze-dried urine samples were resuspended in 10 mM phosphate-buffered saline (PBS) (pH 7.5). Protein concentrations were measured using the BCA Protein Assay Kit (Beyotime, Shanghai, China). Urinary creatinine levels were quantized by alkaline picrate method (Jaffe’s reaction) with creatinine a routine test semi-autoanalyser (Vital Micro 300, Netherlands).

Western blot was used to measure the expression levels of EL. 20 μg of total proteins were used in the experiment. All samples were separated using 4-15% SDS-PAGE (Bio-Rad Laboratories Inc., USA) and transferred onto a PVDF membrane (Bio-Rad Laboratories Inc., USA). The membrane was incubated in 5% milk blocking solution for 2 hours at room temperature. The membrane was incubated with a polyclonal goat anti-human EL primary antibody (1:400; Santa Cruz Biotechnologies, USA) at room temperature for 1 hour, which was washed three times for 5 minutes in phosphate-buffered saline (PBS) and then reacted with a rabbit anti-goat secondary antibody (1:5000; Beyotime, Shanghai, China). For tissues, β-actin antibody (1:1000; Santa Cruz Biotechnologies, USA) was recubated to ensure equal loading. At the end, the membrane was covered completely with an equal amount of enhancer and peroxide solution from an ECL plus Kit (Beyotime, Shanghai, China) for 1 minute, and then all membranes were exposed to the film. The density of the band was quantified using Gel Image System (Tanon, Shanghai, China). The fixed amount of 1 ng purified EL standard (R&D systems, Inc., USA) was used as a positive control for calibration. With consideration the variations in urine formation and excretion, densitometry of EL from urine was expressed relative to urinary creatinine concentrations.

### Immunohistochemistry

For measuring the expression levels of EL in cancer *versus* adjacent noncancerous control tissues, both types of tissues were fixed for 12–16 hours in 4% para-formaldehyde (pH 7.0), embedded in paraffin and cut into 4 μm thick sections. Plus slides were baked at 60°C for 30 minutes. Paraffin was removed using xylene for 30 minutes, and the sections were rehydrated through a series of alcohol solutions before treatment with 1 mM citric acid, pH 6.0, at 100°C for 5 minutes and with 1% H_2_O_2_ for 30 minutes. After being washed with distilled water, sections were incubated at room temperature with 5% porcine sera in PBS for 30 minutes and EL antibody (1:400; Santa Cruz Biotechnologies, USA) diluted in PBS overnight at 4°C, washed three times for 5 minutes in PBS. Bound antibodies were visualized with the color reagent Diaminobenzidine (Bios, Beijing, China). The sections were counterstained with Mayer’s haematoxylin. Two pathologists without knowledge of the patients’ clinical status evaluated all of the stained sections independently. The cells were counted at high magnification in each case (×400), and the percentage of positively staining cells was calculated. The proportion of cells exhibiting EL expression was categorized as follows: 0 = less than 10%; 1 =11% -50%; 2 =51% -75%; 3 = more than 75%. The staining intensity was categorized by relative intensity as follows: 0 = negative; 1 = weak; 2 = intermediate; 3 = strong staining. The proportion and intensity scores were then multiplied to have a total score index. Per statistical analysis, scores lower than 2 were considered negative, and scores of 2 or higher were considered positive.

### Statistical analysis

The chi-square test and Wilcoxon’s signed rank test were used to analyze the expression levels of EL in the paired cancer and noncancerous tissue samples. To analyze the difference of EL expression levels in the serum and urine samples between gastric cancer patients and the healthy controls respectively, Mann–Whitney test was used. The chi-square test was employed to evaluate the relationship between urinary EL expression levels and clinicopathological variables. The discerning power of the expression levels of EL in urine between samples from the cancer and control group was examined using the receiver operating characteristic (ROC) curve and the area under the ROC curve (AUC). All tests were two tailed and a *P*-value < 0.05 was considered statistically significant. All statistical analyses were performed using the GraphPad Prism 5 statistical software.

## Results

### EL expression levels in urine

**Figure 1:**
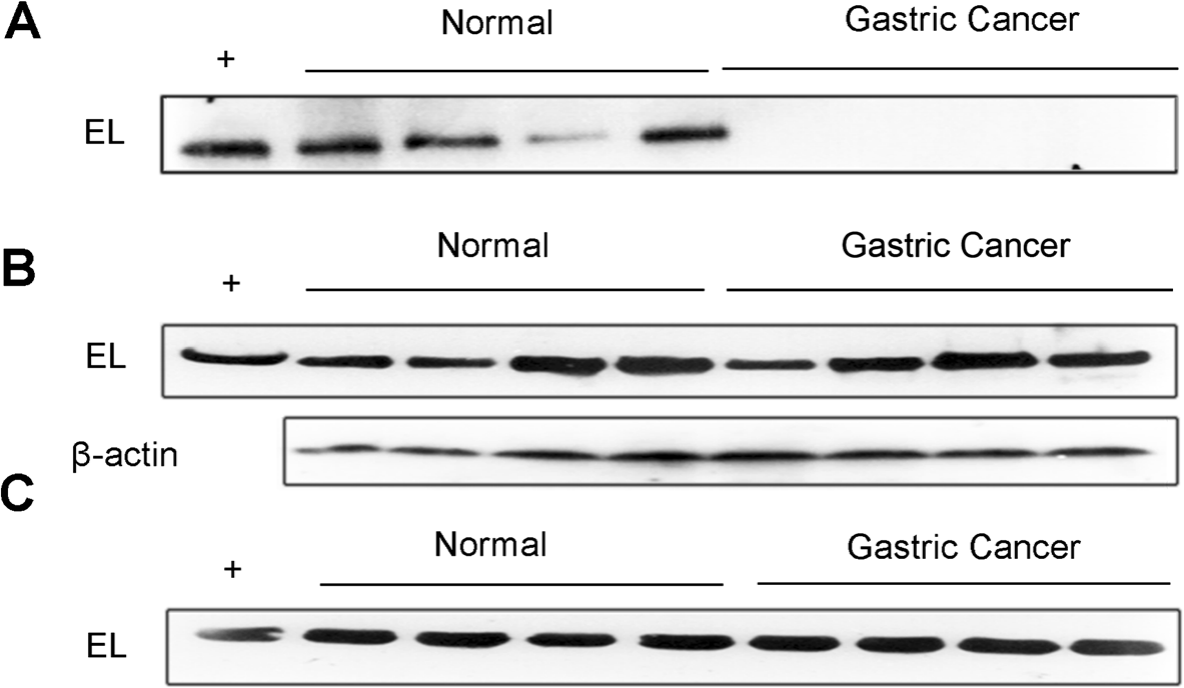
**A representative Western blot analysis of EL protein abundance.** Positive control lane (+) contained the commercial EL standard product, which was used to normalize the signal response across all gels. (**A**), The abundance of EL protein on urine samples; (**B**), EL abundance on cancer tissue samples and matching noncancerous samples from gastric cancer patients with no treatment. The β-actin serves as an internal loading control for estimating the relative protein abundance levels; (**C**), EL protein abundance in serum samples.

**Figure 2:**
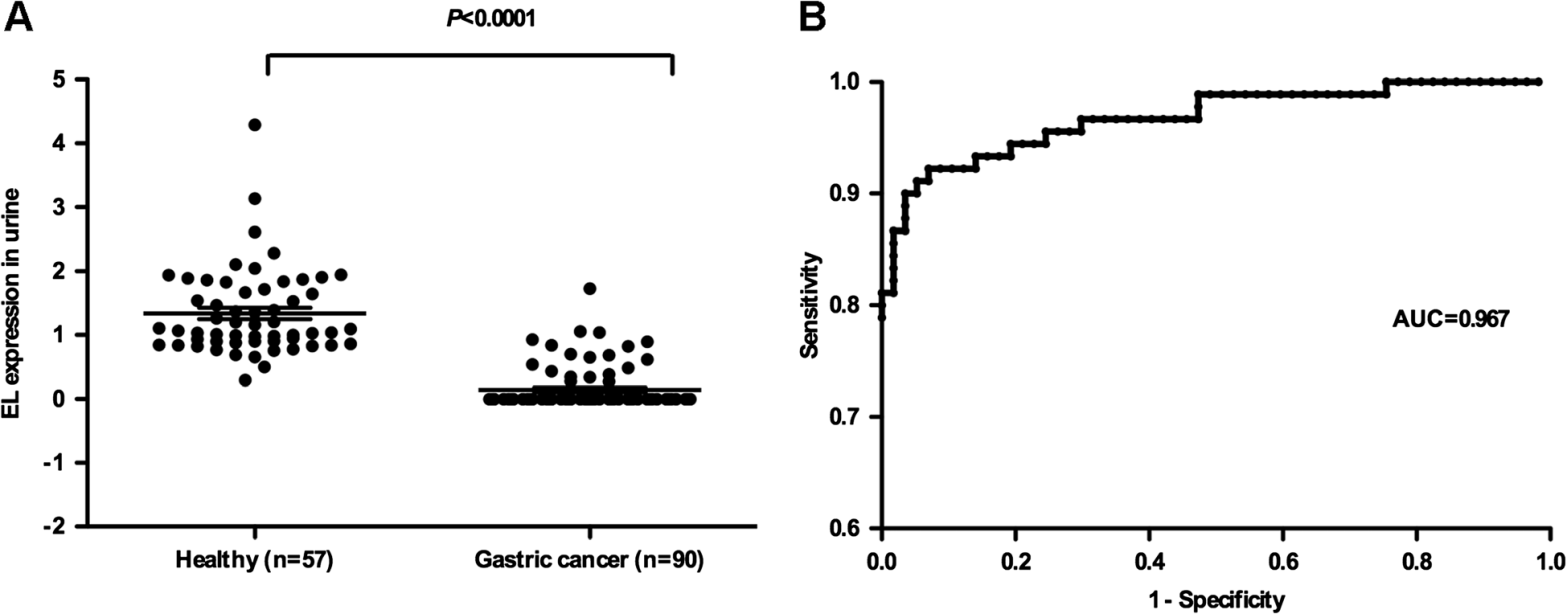
**Urinary EL protein abundances in gastric cancer patients *versus *healthy controls.** Each data point is one individual. (**A**), EL abundance in samples of gastric cancer patients significantly lower than that in samples from healthy controls; (**B**), ROC curves of urinary EL protein. The AUC value was 0.967.

For the semi-quantitative analysis of urine proteins, we used relative EL expression levels with respect to that of the urinary creatinine in each sample to normalize EL expression across different urine samples. We observed a clear separation between the EL expression levels in the urine samples of 90 gastric cancer patients (1.39 ± 0.68) and of 57 healthy volunteers (0.14 ± 0.32). We observed an approximately 9.9 fold average decrease of EL protein levels in the urine samples of gastric cancer patients compared to healthy controls (*P* < 0.0001). The results can be seen in Figure 1A and Figure 2A. We have plotted the ROC curve for the classification accuracy using the EL expression levels across all these urine samples, to provide an overall view of the discerning power of the protein. Note that the AUC provides a widely accepted index for measuring the quality of the underlying classifier with AUC = 1.0 representing perfect classification and AUC = 0.5 representing no separation. EL has achieved an AUC value at 0.967 with the 95% confidence interval (CI) being [0.942-0.993], indicating that urinary EL can serve as a highly promising gastric cancer biomarker for the diagnostic purpose (Figure 2B).

We note that the EL band in Western blot was absent in urine samples of 71 of the 90 gastric cancer patients and all of the 57 healthy volunteers have the EL band in their urine samples. Using absence/presence of the EL band as the cutoff in calling a person having stomach cancer or not, the above data gives rise to a calling sensitivity at 79% [95% CI, 0.690-0.867] and specificity at 100% [95% CI, 0.937-1.000].

We then examined the EL expression levels in urine samples of the gastric cancer patients and its relationship to the various clinicopathological factors, namely gender, histology differentiation, tumor stage, invasion, and metastasis status. No significant statistical relationships between the EL expression levels and any of these factors can be detected. The correlation between urinary EL expression levels and the clinicopathological factors are presented in Table 2.

**Table 2:** Correlation between urinary EL expression and clinicopathological features

**Clinicopathological (n)**	**EL absent (%)**	**EL present (%)**	***P***
	**n = 71**	**n = 19**	
Gender			
Male, (67)	51 (72)	16 (84)	0.27
Female, (23)	20 (28)	3 (16)	
Histological type			
High or moderate, (43)	31 (44)	12 (63)	0.13
Poor or undifferentiated, (47)	40 (56)	7 (37)	
Lauren classification			
Intestinal, (39)	29 (41)	10 (53)	0.36
Diffuse, (51)	42 (59)	9 (47)	
Tumor stages			
Ι and ΙΙ, (31)	22 (31)	9 (47)	0.18
ΙΙΙ and ΙV, (59)	49 (69)	10 (53)	
Depth of invasion			
T1 and T2, (30)	21 (30)	9 (47)	0.14
T3 and T4, (60)	50 (70)	10 (53)	
Node status			
Absent, (29)	23 (32)	6 (32)	0.95
Present, (61)	48 (68)	13 (68)	
Metastasis status			
M0 (71)	55 (77)	16 (84)	0.52
M1 (19)	16 (23)	3 (16)	

**Figure 3:**
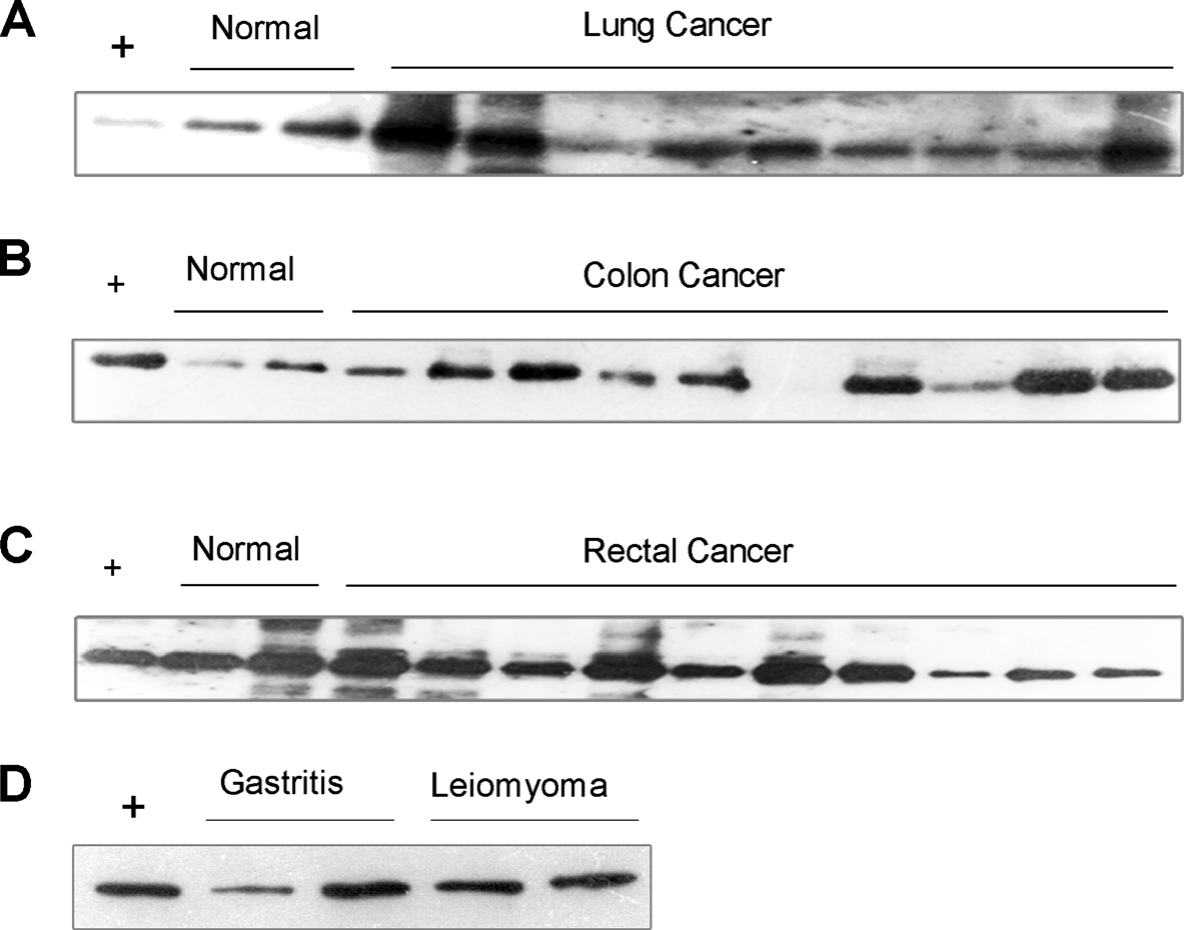
**EL abundances in urine samples of other cancer types and benign lesions.** (**A**), Nine lung cancer samples; (**B**), Ten colon cancer samples; (**C**), Ten rectal cancers samples; (**D**), Two gastritis and two gastritic leiomyoma samples. (+) The positive control lane.

We have also checked if EL is specific to gastric cancer. As an initial effort, we have examined the EL expression levels in urine samples of three other cancer types (lung, colon and rectum cancers) and benign lesions (gastritis and gastric leiomyoma). The test was done on urine samples of 9 lung cancer, 10 colon cancer, 10 rectum cancer, 2 gastritis, and 2 gastric leiomyoma patients. Clearly we can see that 9 out of 9, 9 out of 10, 10 out of 10, 2 out of 2, and 2 out 2 samples have the EL band in the urine samples of lung cancers, colon cancers, rectum cancer, gastritis and gastric leiomyoma patients, respectively (Figure 3). While this result does not guarantee that the lack of EL in urine is an indicator of only gastric cancer, it does strongly suggest that EL is highly specific. Larger tests will be done in our follow-up study, which will involve substantially more patients of other diseases possibly related to gastric cancer, such as pancreatic cancer and esophagus cancer.

### EL expression levels in tissues and sera

The study indicates that the EL protein is absent in gastric cancer urine samples. Considering that urine contains both protein secreted from urothelium as well as proteins from the plasma or cell lysis, we have also examined the EL expression levels in tissue or serum of gastric cancer patients, aiming to infer the reasons for this observed absence. Western blot was carried out to measure the EL expression levels in cancer tissue samples and matching noncancerous samples from 12 untreated gastric cancer patients (Figure 1B). No significant difference in the expression levels of EL was observed between the gastric cancer tissue samples and the matching noncancerous samples (Wilcoxon’s signed rank test, *P* =0.90).

**Figure 4:**
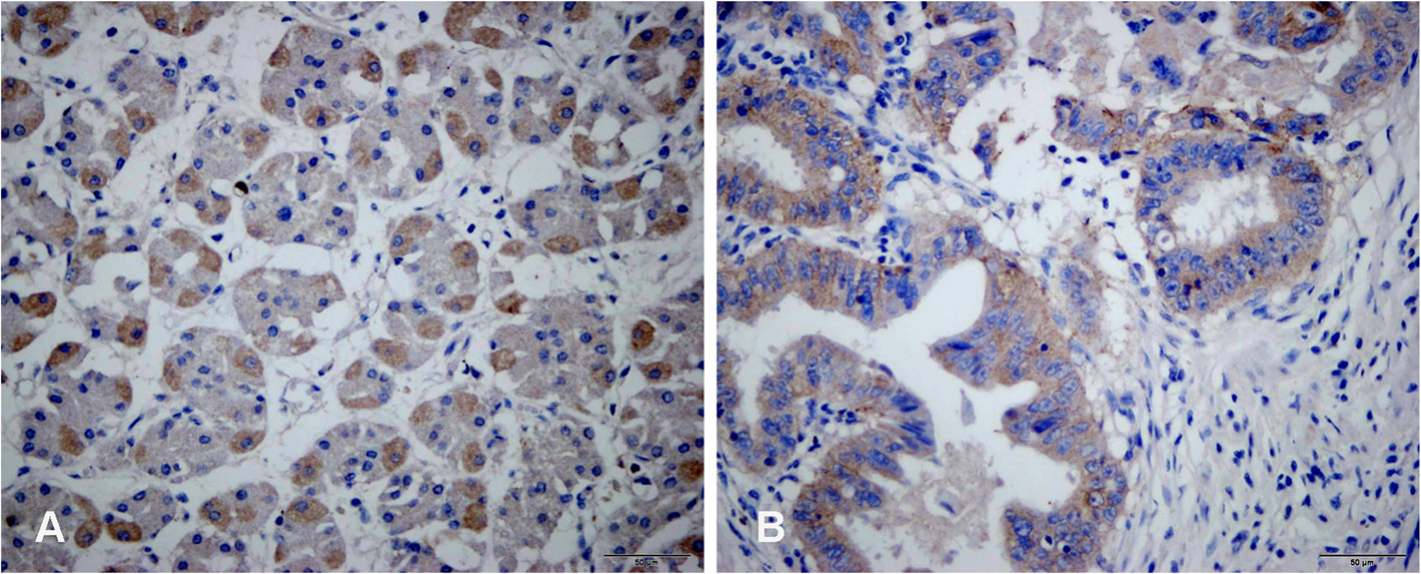
**Immunohistochemical analysis of EL in tissues of gastric cancer and matching noncancerous samples.** The brown granules of EL predominantly appeared in cytoplasm. Adjacent noncancerous tissues stained with (**A**) (×400). Cancer tissues stained with (**B**) (×400).

In immunohistochemical staining experiments, we found that the brown granules predominantly appeared in the cytoplasm (Figure 4). Positive expression of the EL protein was found in 58% (7/12) of gastric cancer and in 67% (8/12) of gastric adjacent noncancerous samples (Table 3). There was no difference in the expression levels of this protein between the gastric cancer and the matching noncancerous tissues (chi-square test, *P* =0.67). We also examined any possible relationship between the EL expression levels and potentially relevant clinicopathological factors mentioned earlier. No relationship was detected. The results are presented in Table 4.

**Table 3:** EL expression in gastric cancer and adjacent noncance rous samples

	***n***	**The expression of EL protein**		***P***
		**Negative**	**Positive**	
**Adjacent control**	12	4(33%)	8 (67%)	0.67
**Gastric cancer**	12	5(42%)	7 (58%)	

**Table 4:** EL expression and clinicopathological factors in gastric cancer tissues

**Clinicopathological**	***n***	**EL**		***P***
		**Negative**	**Positive**	
Gender				
Male	6	2	4	0.56
Female	6	3	3	
Age (year)				
≤50	3	1	2	0.74
>50	9	4	5	
Lauren classification				
Intestinal	5	3	2	0.28
Diffuse	7	2	5	
Histological type				
Highly differentiated	1	1	0	0.36
Moderately differentiated	4	2	2	
Poorly differentiated	7	2	5	
Tumor stages				
I and II	2	1	1	0.79
III and IV	10	4	6	
Depth of invasion				
T1 and T2	5	2	3	0.92
T3 and T4	7	3	4	
Lymph node metastasis				
Positive	3	1	2	0.74
Negative	9	4	5	

We then measured the EL expression levels in sera of the 12 gastric cancer patients *versus* those of 12 healthy people (Figure 1C). It can be seen that there is no clear distinction between the cancer samples and the healthy control (Mann–Whitney test, *P* = 0.79). We note that the EL expression levels have a wide range distribution in both sets of samples.

## Discussion

In our previous study, we developed a novel computational method and applied it to predict that EL could potentially serve as a highly promising diagnostic urinary marker for gastric cancer. Here, we have further confirmed this prediction on larger sample set, and in addition we discovered that the EL protein is highly specific to gastric cancer.

EL is a new member of the triglyceride lipase gene family, and has high sequence homology with lipoprotein lipase (LPL) (45%), hepatic lipase (HL) (41%) and pancreatic lipase (PL) (21%) [19,20]. EL has primarily a phospholipase and has some triglyceride lipase activity, which has an important role in plasma high-density lipoproteins metabolism and atherosclerosis development [21-25]. Several studies have shown that LPL plays an important role in carcinogenesis, including colorectal and pancreatic cancers, and lung cancer [26,27]. However, EL has not been reported to be associated with any cancer except for testicular germ cell tumors, while the mechanism there is unclear [28].

The discovery that the expression levels of EL is significantly reduced in urine of gastric cancer patients, while showing no difference between the corresponding serum samples as well as tissue samples is very intriguing. One potential reason could be due to the properties of the glomerular filtration system. It is known that plasma proteins were filtered though the glomeruli on the basis of their sizes, charges and structure shape [29]. Small and positively charged molecules were more easily filtered into urine than large and negatively charged proteins. The sequence of EL has a number of positively charged clusters [30], which may be a reason that it can be filtered out in healthy people’s urine. However, the microenvironment of the cancer cells tend to be more acidic [31], which may potentially change positively charged clusters of ELs to negative ones, hence preventing the molecules from being filtered into the urine. Certainly, the precise reason is yet to be understood, and warrants further studies.

Urine is an ideal non-invasive source for cancer detection. However, it should be noted that urine has a high degree of variability in protein concentrations throughout a day, which can be influenced by various factors (age, diet, and collection time). For this reason, it is a key to normalize the protein concentration in urine when measuring the expression levels of protein. Therefore, we used the relative EL expression levels with respect to that of the urinary creatinine in each sample to normalize EL expression levels across different urine samples. The results of this study suggest that the loss of urinary EL expression can provide a preliminary indication of gastric cancer during large-scale screening and more direct examinations such as gastroscopy and pathology test on the biopsy sample will be needed for the final diagnosis. Many studies report that diagnostic biomarker may be a useful prognostic and survival indicator for gastric cancers [32-34]. It was not only to predict the prognosis information of cancer patients, but also to provide the treatment strategy for the physician. So we examined the correlation between EL expression levels and the clinicopathological characteristics in gastric cancer. Although EL makes a promising diagnostic marker for gastric cancer, we did not find any strong relationships between the EL expression level and the tumor’s prognostic clinicopathological characteristics such as tumor type, invasion and TNM staging. In addition, because of the short term of study and lack of regular follow up of patients, we can not assess survival rate in this study. The limited existing data showed that EL expression can not be used as a useful prognostic and survival indicators for gastric cancer. The next study, we aimed to investigate the EL protein expression in larger numbers of patients with different tumors and its follow up studies, and to explore the exactly mechanism of EL’s role in the carcinogenesis of gastric cancer.

## Conclusion

In conclusion, although it is not linked to tumor stage or grade, urinary EL could be a highly promising gastric cancer biomarker that may be applicable to large-scale screenings with high diagnostic fidelity.

## Abbreviations

EL: Endothelial lipase; ROC: Receiver operating characteristic curve; AUC: The area under the ROC curve

## Competing interests

The authors declare that they have no competing interests.

## Authors’ contributions

XYD and GQW drafted the manuscript. ZHN and JC helped perform the evaluation. XYD and AC carried out the experiments. JS and WQZ collected clinical data and participated in the evaluation of the immunohistochemistry. QY, YX and FL contributed to the conception and design of the study. All authors read and approved the final manuscript.
